# Single prolonged stress in mice: interactions between alcohol drinking, negative affect, and fear learning

**DOI:** 10.1007/s00213-025-06968-8

**Published:** 2025-11-24

**Authors:** Aditi B. Buch, Ava L. Shipman, Nicolas J. Azzarello, Annie W. Zhou, Samuel W. Centanni

**Affiliations:** Department of Translational Neuroscience, Wake Forest University School of Medicine, Medical Center Boulevard, Winston-Salem, NC NC 27103 USA

**Keywords:** Stress, Alcohol, Negative affect, Startle, Fear conditioning, SPS, Binge-like consumption, Volitional drinking, Sex differences

## Abstract

**Rationale & objective:**

Exposure to traumatic stressors can have detrimental effects on one’s well-being. Alcohol is often used as a coping mechanism to alleviate stress, leading to the development of alcohol use disorder (AUD). Furthermore, sex dimorphisms in stress and AUD alter their progression and sustenance. To investigate these interactions for effective treatment strategies, validated and exhaustive preclinical models are necessary. Here, we designed a comprehensive study that examines how traumatic stress influences ethanol drinking patterns and PTSD-like phenotypes in male and female mice with Single Prolonged Stress (SPS).

**Methods:**

Male and female C57BL/6 J mice underwent SPS, a model of traumatic stress consisting of a series of consecutive stressors, followed by a 7-day stress incubation period. Mice then underwent a series of tests for aversive state, fear discrimination, and drinking patterns during continuous and limited ethanol access.

**Results & conclusions:**

SPS selectively increased negative affect and startle responses in male mice, while females were unaffected. SPS disrupted discrimination adjustment between fear and safety cues during extinction, while ethanol exposure attenuated overall fear responses. SPS maintained baseline sex differences in consumption. Furthermore, female consumption increased when access was provided prior to and continuously throughout SPS and fear conditioning, contrasting with groups exposed to ethanol afterwards. In summary, we identified a distinct sex specific relationship between traumatic stress, fear memory, and the timing of ethanol consumption. We highlight SPS as a robust translational model for exploring the sex-specific neurobiological mechanisms driving traumatic stress-induced affective disturbances.

**Supplementary Information:**

The online version contains supplementary material available at 10.1007/s00213-025-06968-8.

## Introduction

Stress is a common part of our lives, and responding to everyday stressors is imperative to a healthy lifestyle. Further, the intensity, frequency, and type of stressor can lead to maladaptive coping mechanisms and inability to respond to the stress appropriately. Repeated traumatic experiences increase the probability of developing stress disorders such as Post Traumatic Stress Disorder (PTSD) (Kube et al. 2023; Herman 1992) and substance use disorders such as Alcohol Use Disorder (AUD) (Ruglass et al. 2017). While clinical studies of the patient population with PTSD are very complex and provide insight into the symptomology, they cannot probe for biological modulations caused by traumatic stress. Thus, preclinical models of traumatic stress serve as an invaluable tool to characterize behavior and the mechanisms that drive it. Defining new models that encompass variable components of stress and AUD-related behavior is an essential step toward developing new treatments and diagnostic strategies.

Single Prolonged Stress (SPS) is a validated preclinical model of PTSD (Liberzon et al. 1997) that relies on acute, multimodal, and sequential experience of psychological, physical, and pharmacological stressors. SPS accurately models a key feature of clinical trauma, the delayed onset of negative emotional symptoms, as rodents display behavioral changes only after a 7-day undisturbed “consolidation period” immediately after SPS (Fang et al. 2018). Furthermore, this consolidation period enhances the duration of these phenotypes to reveal PTSD-like pathology, better modeling a sustained PTSD response seen in patients (Richter-Levin et al. 2019). Several studies in rats have used SPS to model affect-like behavioral changes and HPA-axis dysfunctions that are foundational of PTSD symptomology (Pitcairn et al. 2024; Guan et al. 2022; Liu et al. 2016; Han et al. 2014; Ganon-Elazar and Akirav 2012; Liberzon et al. 1997, 1999). Adapting SPS for mice, with the only modification being the inclusion of “predator odor” (Perrine et al. 2016), successfully produced similar phenotypes to those observed in rats mentioned above (Marques et al. 2023; Perrine et al. 2016). SPS offers an excellent option for modeling traumatic stress; however, several gaps in this endeavor remain, particularly in mice, which we aim to address in this study.

Although there is compelling evidence suggesting that stress is a risk factor for AUD (Keyes et al. 2012), alcohol consumption following SPS exposure has been an understudied area. Chronic intermittent ethanol exposure in mice following SPS resulted in increased vapor-induced ethanol intake, but not volitional intake (Piggott et al. 2020). Similarly, Pitcairn et al. (2024) report no changes in the volitional drinking model, two-bottle choice (2-BC), following SPS in rats. Investigating multiple drinking paradigms, such as DID and 2-BC with SPS in mice, can reveal diverse drinking dynamics resulting from traumatic stress in these preclinical studies. Another benefit of adapting SPS for mice is that rats are limited in procedures involving genetic manipulations, such as transgenics, optogenetics, and *in vivo* recordings, compared to mice (Bryda 2013).

Another key PTSD symptom is a deficit in fear extinction, leading to abnormal fear responses. SPS induces deficits in extinction after contextual fear conditioning in rats (Yamamoto et al. 2008), implying that SPS can model these dysregulated fear responses. The deficits seen in extinguishing fear memories in PTSD patients are thought to motivate increased alcohol consumption, leading to AUD (Dell’Aquila and Berle 2023). To address this intricate relationship, we have established a comprehensive behavioral model with SPS to explore the effects of traumatic stress on fear memory and ethanol consumption. In this study, the use of SPS as a traumatic stressor and fear conditioning (FC) as a measure of the three stages of fear memory (fear conditioning, fear recall, and extinction recall) facilitated a robust exploration of the impact of traumatic stress on fear memory and ethanol consumption. To account for populations that are exposed and have access to alcohol prior to and during stressors, two-bottle choice (2-BC), a continuous home cage drinking model, was used at different timepoints for each group and compared. This comprehensive model enabled examination of the intricate relationship between traumatic stress, fear memory, and ethanol consumption in mice to model clinical populations. This further emphasizes the potential for mouse SPS to model complex clinical PTSD phenotypes, especially predisposition to alcohol use.

In addition to the complex phenotypes seen from traumatic stress, there are also sex differences that require consideration. Women report stress, anxiety, and depression at a much higher rate than men (McLean et al. 2011; Bruce et al. 2005; Kessler 1994; Regier et al. 1990; Angst and Dobler-Mikola 1985), suggesting females may be more vulnerable to stress disorders. Growing evidence in recent years points to an increase in the prevalence of AUD in women compared to men (Salazar and Centanni 2024; Grucza et al. 2018; Grant et al. 2016; White et al. 2015), indicating that women tend to misuse alcohol to cope with psychological stressors and face higher rates of relapse driven by stress-induced negative emotions (Kerr-Corrêa et al. 2007; Erol and Karpyak 2015; Greenfield et al. 2010; Guinle 2020; Peltier et al. 2019). Given the disproportionate number of women affected by stress-related behavioral impairments, it is essential to consider how traumatic stress might differently affect males and females in preclinical research. Despite this known sexual dimorphism, most studies using SPS rely on male rats or mice. Thus, further studies are needed to obtain a holistic understanding of how SPS alters stress responses, such as fear extinction, and ultimately drives affect-like drinking behaviors in both males and females. Conducting these experiments in mice will provide a foundation for future studies aimed at dissecting the neurophysiology and circuitry of traumatic stress-induced changes in behavior.

This study addresses the need for a validated preclinical model of PTSD in mice, particularly those considering crucial variables like sex and ethanol exposure time windows. Our work aims to address this gap by exploring Single Prolonged Stress (SPS) in mice, and its relationship with alcohol drinking, negative affect-like behavior, and fear learning- all of which are intertwined with phenotypes of PTSD. We demonstrate that SPS elicits modality- and sex-specific PTSD-like phenotypes and temporal drinking patterns in mice. By meticulously characterizing SPS as a robust and translational model, we characterize a crucial tool for investigating the sex-specific neurobiological mechanisms that drive traumatic stress-induced affective disturbances and alcohol misuse, paving the way for more nuanced and effective therapeutic interventions.

## Methods

### Subjects

All experiments were conducted in accordance with the National Institutes of Health Guide for the Care and Use of Laboratory Animals and approved by the Wake Forest University Institutional Animal Care and Use Committee. A total of 124 adult male and female mice from C57BL/6 J background strain (The Jackson Laboratory; Bar Harbor, ME) were used. All animals were acclimated in standard group housing for one week upon arrival and were handled for at least a week prior to an experiment. All animals in the drinking experiments and fear conditioning experiments were maintained on a 12 h reverse light/dark cycle (lights on at 1800 h) and animals used to probe for negative affect were maintained on 12 h light/dark cycle (lights on at 0600 h) under controlled temperature (20–25 °C) and humidity (30–50%) levels. Mice were given ad libitum access to food and water. Mice were acclimated in a group house setting but switched to single housing immediately after completing SPS.

### Single prolonged stress (SPS)

We adapted the SPS procedure detailed by Perrine et al. (2016). Mice randomly assigned to the SPS group first underwent 2 h of restraint stress (RS), during which they were placed in BD Falcon 50 ml conical tubes with a screw-on top (with air holes located approximately 1/2 cm apart). Immediately following RS, animals underwent 7 min of grouped forced swim (FS) (4–5 mice/swim) in a 5-L beaker (diameter: 20 cm) filled to ~ 4 L (depth: ~ 19 cm) with room temperature water (23-27ºC). Mice were briefly dried with paper towels after FS and then placed in a cage with soiled rat bedding from sex-matched rats to induce a predator odor (PO) stress, a physiological and psychological stressor. Following 15 min of PO, mice were placed in a clean cage with a ventilated lid. Here, they were exposed to diethyl ether (Et) until loss of consciousness. Every minute, a cotton ball soaked with 3 ml of ether was added to the cage. Mice were observed for loss of righting reflex, upon which they were moved back to clean home cages and singly housed. SPS animals were left undisturbed for seven days following initial exposure, allowing behavioral phenotypes to emerge (Liberzon et al. 1997). On SPS day, control animals were placed in a room behind where SPS occurred. At the end of the SPS day, control animals were also singly housed and left undisturbed for seven days before any testing began.

### Social interaction (SI)

Mice were placed in a 3-chamber box (Triad Plastics, 2 ft × 1.5 ft) and given 5 min to habituate to all the chambers. After 5 min, the test mice were returned to their homecage while a novel mouse of the same sex and approximately the same age was placed under a wire cup in either chamber. The chamber where the novel mouse was placed was alternated between each trial to prevent any biases. The test mouse was then placed back into the center of the 3-chamber box and allowed 10 min to explore. During the habituation phase, time spent on each side of the 3-chamber box was recorded. The time spent on each side of the 3-chamber box and the total time spent directly interacting with the novel mouse during the testing phase were noted by a blinded experimenter after the experiment, via a video recording.

### Elevated plus maze (EPM)

Mice were placed in the center of a standard elevated plus maze (EPM; Triad Plastics, Winston-Salem, NC, USA; Arm Length: 35cm, Arm Width: 5cm, Wall Height: 20cm, Stand Height: 61cm, Edge Bumper: 1cm) containing two open arms and two closed arms for 5 min. The open-arm light measured 600 lx, while the closed light measured 165 lx. A blinded experimenter scored the time spent in the open arms via a video recording after the experiment.

### Novelty suppressed feeding test (NSFT)

The novelty suppressed feeding test (NSFT) was conducted as previously described (Holleran et al. 2016; Centanni et al. 2019; Britton and Britton 1981). Mice were food-restricted for the 48 h leading up to the test. Food access was granted for a 2-h period, 23–25 h prior to NSFT. Forty-eight hours into NSFT, mice were placed in an open arena (San Diego Instruments, San Diego, CA, USA; 50 cm x 50 cm) with bedding and a food pellet at the center of the brightly lit apparatus (300 lx). Latency to eat was measured as the amount of time elapsed before the subject took a bite of the food pellet. The latency to eat was assessed in real time by the experimenter and confirmed through video recording after the experiment. Mice were removed immediately after the first bite and placed back into their home cage with a pre-weighed food pellet. After 10 min, the food pellet was reweighed to determine home cage consumption.

### Startle tests

In order, the three startle tests were the Airpuff, Acoustic, and Footshock tests. All testing was conducted using the SR Lab Startle Response system (San Diego Instruments, San Diego, USA). Mice were placed in a ventilated chamber containing a stabilimeter, which consisted of a Plexiglas-mounted tube on a Plexiglas base. All tests were carried out with the startle box lights turned on (230 lx). Each run started with 3 min of habituation, followed by the startle stimuli described below. Each stimulus intensity was presented three times in a randomized order, with inter-stimulus intervals ranging from 20 to 40 s. Each startle was transduced by an accelerometer located under the Plexiglas base and recorded as 1000-ms readings, beginning at the onset of each startle stimulus and lasting 10 s. Max peak startle amplitude and latency to max peak were evaluated for each stimulus intensity in each startle using the San Diego Instruments Wave Form Analysis software.

#### Airpuff startle

An air puff startle session consists of 3 min of habituation followed by three runs of a single airpuff startle at one intensity (30 psi), accounting for three total startles per mouse. A full testing session of air puff startle for each mouse lasted about 5 min.

#### Acoustic startle

An acoustic startle session consists of 3 min of habituation followed by three runs of 3 separate acoustic startles at different intensities, totaling nine startles per mouse. The three acoustic intensities were 90 dB, 95 dB, and 105 dB. Stimuli were presented in that order of intensity for all three runs. Each of the three runs was separated by an intertrial interval of 3 min. A full testing session of acoustic startle for each mouse lasted about 15 min.

#### Footshock startle

A foot shock startle session consists of 3 min of habituation followed by three runs of 6 separate foot shock startles at different intensities, accounting for 18 total startles per mouse. The six shock intensities were 0.2 mA, 0.4 mA, 0.05 mA, 0.6 mA, 0.1 mA, and 0.8 mA; and were given in that order of intensity for each run to maximize randomization. Each of the three runs was separated by an intertrial interval of 3 min. A complete testing session of foot shock startle for each mouse lasted about 20 min.

To evaluate the various behavioral parameters tested across these different assays, a comprehensive Emotionality Score (Guilloux et al. 2011) was calculated for each animal. Z-scores were calculated with the following formula:

$$z=\frac{(X-\mu )}{\sigma }$$

where X is the individual data point from each observed parameter and $\mu$ and $\sigma$ are the mean and standard deviation for the control animals, respectively were calculated. Z-scores were averaged for each animal across every behavioral parameter to get an Emotionality Score.

### Drinking in the dark (DID)

Drinking in the Dark (DID) was conducted as previously described (Rhodes et al. 2005; Thiele et al. 2014). DID began 3 h into the dark cycle. Water bottles were replaced with a single bottle containing 20% EtOH. Mice were given access to the EtOH bottle for 2 h a day for three consecutive days (Monday-Wednesday), and on the fourth day (Thursday), mice had access to the EtOH bottle for 4 h. After each drinking session, the EtOH bottles were weighed to assess volume consumption and replaced with their corresponding water bottles. Following the 4-h session, the mice remained on water for three consecutive days (Friday to Sunday). Mice underwent three successive cycles of DID lasting 3 weeks total.

### Volitional continuous access two-bottle choice drinking (2-BC)

Two bottles were placed in the animal’s home cage and were continuously available for 24 h a day. One bottle contained water, and the other bottle was filled with 10% EtOH. Mice and bottles were weighed for volume consumption on three days a week: Mondays, Wednesdays, and Fridays.

### Fear conditioning (FC)

We utilized two versions of fear conditioning; the first (FC1) was a standard differential fear conditioning paradigm adapted from Haufler et al. (2013) and used with the EtOH + FC group. A second modified version (FC2) was used on the other groups to allow us to collect more data on fear recall and extinction behavior. FC2 was adapted from Klein et al. (2021). In all groups, two distinct contexts, Context A and Context B, were used across sessions to facilitate discrimination and reduce contextual generalization. Context B differed from Context A by the addition of peppermint extract as an olfactory feature. Mice had a habituation period in the context each session for 120 s before any trials began. Differences between the two are denoted below as FC1 and FC2.

Habituation: Mice were habituated to both Context A and B for 20 min, with sessions separated by at least 3 h. Animals in FC1 were not habituated to the tones on this day. In FC2, animals were exposed to four presentations each of the conditioned stimulus (CS + ; 80 dB; 5 kHz; 20 s) and the control stimulus (CS-; white noise; 20 s). Intertrial intervals (ITIs) ranged from 30 to 130 s.

#### Conditioning

FC1: In Context A, mice were first habituated to the auditory tones and received five presentations of CS + (80 dB, 10 s) and CS- (60 dB, 10 s). After, mice underwent fear conditioning, during which the CS + was paired with a mild footshock (unconditioned stimulus, US; 0.8 mA, 1 s) that co-terminated with the last second of the tone to induce a fear cue. The CS- was presented without any shock to serve as a safety cue. Each stimulus was presented five times with ITIs ranging from 2 to 3 min.

FC2: In Context A, mice underwent fear conditioning, during which the CS + was paired with a mild footshock (unconditioned stimulus, US; 0.6 mA, 2 s) that co-terminated with the last 2 s of the tone to induce a fear cue. The CS- was presented without any shock to serve as a safety cue. Each stimulus was presented five times with ITIs ranging from 30 to 120 s.

#### Contextual fear

On day 3 for FC1, mice were returned to Context A for 10 min and did not receive any sounds to extinguish contextual fear.

#### Fear recall and extinction

For FC1, recall and extinction were conducted in a single session. Mice were placed in Context B and exposed to each tone 20 times, where the first 10 trials served as Fear Recall and the last 10 trials served as Extinction Recall. ITIs varied between 2 and 3 min.

For FC2, Fear Recall and Extinction were separated across three days in FC2. For Recall, mice were returned to Context A and presented with four trials each of CS + and CS-, in the absence of the US, to assess fear memory recall. ITIs ranged from 30 to 60. Two days of Extinction training followed. Mice were placed in Context B and exposed to four non-reinforced presentations of both CS + and CS- per day to facilitate extinction learning. ITIs varied between 30 and 90.

#### Extinction recall

To assess extinction memory in FC2, mice were tested in Context B with four non-reinforced presentations of CS + and CS-. ITIs ranged from 40 to 50 s.

### Analysis of freezing behavior

#### Normalized freezing scores

Freezing behavior was quantified as the cumulative duration of immobility (in seconds) during each 20-s CS + and CS − auditory cue presentation using EthoVision XT. For each animal, freezing during the conditioning, fear recall, and extinction recall sessions was normalized to its respective baseline freezing during the habituation session for the same cue type (CS + or CS −). Normalization was performed using the following formula:

$$\normalsize \begin{aligned}&NormalizedFreezing\left(CS type\right)=\\&\frac{\left[Freezing\left(CS type,DayX\right) - Freezing\left(CS type,Habituation\right)\right]}{\left[Freezing\left(CS type,Habituation\right)\right]+\in } \end{aligned}$$

Day X refers to any post-habituation test session (e.g., Conditioning, Fear Recall, and Extinction Recall). *ϵ* = *1 s* to prevent division by zero and stabilize estimates for animals with low baseline freezing. This value was selected based on a distributional analysis, which showed that ~ 25% of mice froze for less than 1 s at baseline.

Discrimination Index (DI): To assess cue discrimination between CS + and CS-, a DI was calculated per animal using the normalized freezing values:

$$DI=\frac{\left(\frac{\left(CS+\right) - \left(CS-\right)}{\left(CS+\right) + \left(CS-\right)}\right)}{Total Cue Duration} \times 100{\%}$$

DI values were calculated for each session and used to compare group, sex, and day-specific effects on cue discrimination. Transforming the DI scores to a percentage scale enabled a more straightforward interpretation of the magnitude and direction of cue discrimination, where positive values indicate more freezing towards CS + , negative values indicate more freezing towards CS-, and values near zero suggest no cue discrimination. Given the ratio and normalization procedure, some extreme values could exceed the bounds of a valid percentage scale. In such cases, DI values were capped at ± 100, representing the maximum and minimum discrimination scores possible given the 20 s tone duration.

### Timing of ethanol exposure, SPS, and FC experimental groups

The following groups investigated the effects of the timing of ethanol exposure, prior stress, and fear on ethanol consumption in males and females (Fig. 4). The last two weeks of ethanol consumption were compared between the groups. These groups were also used to explore ethanol exposure and prior stress on fear learning (Fig. 5).

#### FC group

Mice underwent fear conditioning and then had continuous access to 10% ethanol (2-BC) for two weeks.

#### SPS + FC group

Mice underwent SPS and after their undisturbed week underwent fear conditioning. After the last fear conditioning day, mice had two weeks of continuous access to 10% ethanol (2-BC).

#### EtOH + FC group

Mice had four weeks of continuous access to 10% ethanol (2-BC) before undergoing fear conditioning while still having ethanol access in their home cages. Mice continued 2-BC drinking after fear conditioning for two more weeks.

#### EtOH + SPS + FC group

Mice had four weeks of continuous access to 10% ethanol (2-BC) before undergoing SPS and continued having ethanol access in their home cages during the undisturbed week. Afterwards mice, with continual 2-BC drinking, underwent fear conditioning and then continued having ethanol access for two more weeks.

### Statistical analyses

Statistical analyses were conducted using GraphPad Prism (v10.4.1). Specific tests performed on each experiment are listed in the results section. A value of *p* < 0.05 was considered statistically significant. All data are presented as mean ± SEM.

## Results

### Single prolonged stress selectively induces negative affect in male mice during the novelty suppressed feeding test.

**Fig. 1:**
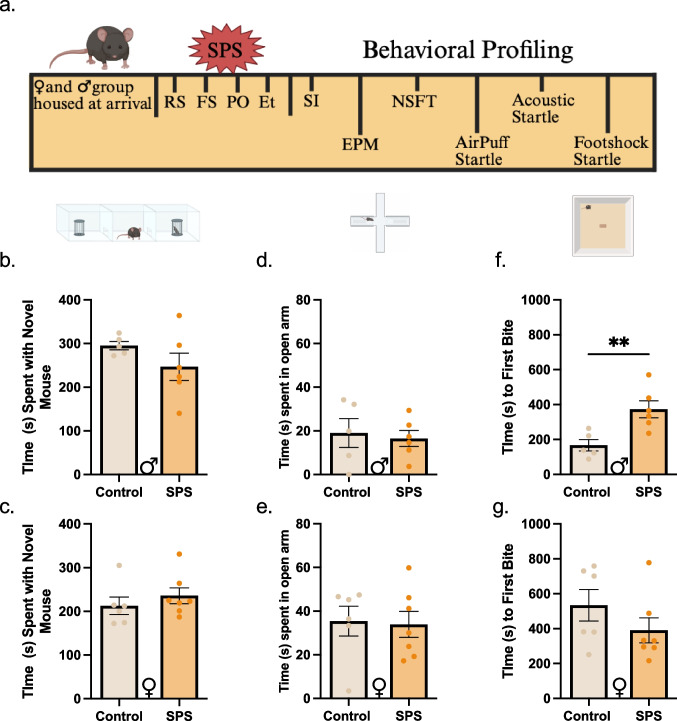
SPS exposure selectively induced negative affect in male mice

Clinical and preclinical studies suggest that traumatic stress has a strong relationship with increases in affect-like behaviors (Galatzer-Levy et al. 2013; Weera et al. 2023). To characterize the effects of SPS on affective behavior in mice, males and females underwent SPS followed by a one-week incubation period that is required for behavioral phenotypes to arise in both mice and rats (Fang et al. 2018; Perrine et al. 2016; Liberzon et al. 1997) (Fig. 1a). Animals then underwent a series of tests validated to measure aspects of negative affect-like behaviors. During Social Interaction (SI), SPS had no impact on time spent with the novel mouse, regardless of sex (Student’s t-test: males; t_(9)_ = 1.358, *p* = 0.2077; females; t_(11)_ = 0.8484, *p* = 0.4143; Fig. 1b-c). Likewise, time spent in the open arms on Elevated Plus Maze (EPM) was similar in SPS and control mice (Student’s t-test: males; t_(9)_ = 0.3478, *p* = 0.7360; females; t_(11)_ = 0.1633, *p* = 0.8733; Fig. 1d-e). When mice underwent Novelty Suppressed Feeding Test (NSFT), SPS led to a higher latency to eat in the males (Student’s t-test: t_(9)_ = 3.384, *p* = 0.0081; Fig. 1f), while no differences were noted in the latency to eat in the females (Student’s t-test: t_(11)_ = 1.263, *p* = 0 0.2327; Fig. 1g). Thus, SPS induced avoidance behavior in male mice on the NSFT, but not on the EPM, suggesting that this effect is modality-specific.

### Single prolonged stress has specific startle effects in male mice.

**Fig. 2:**
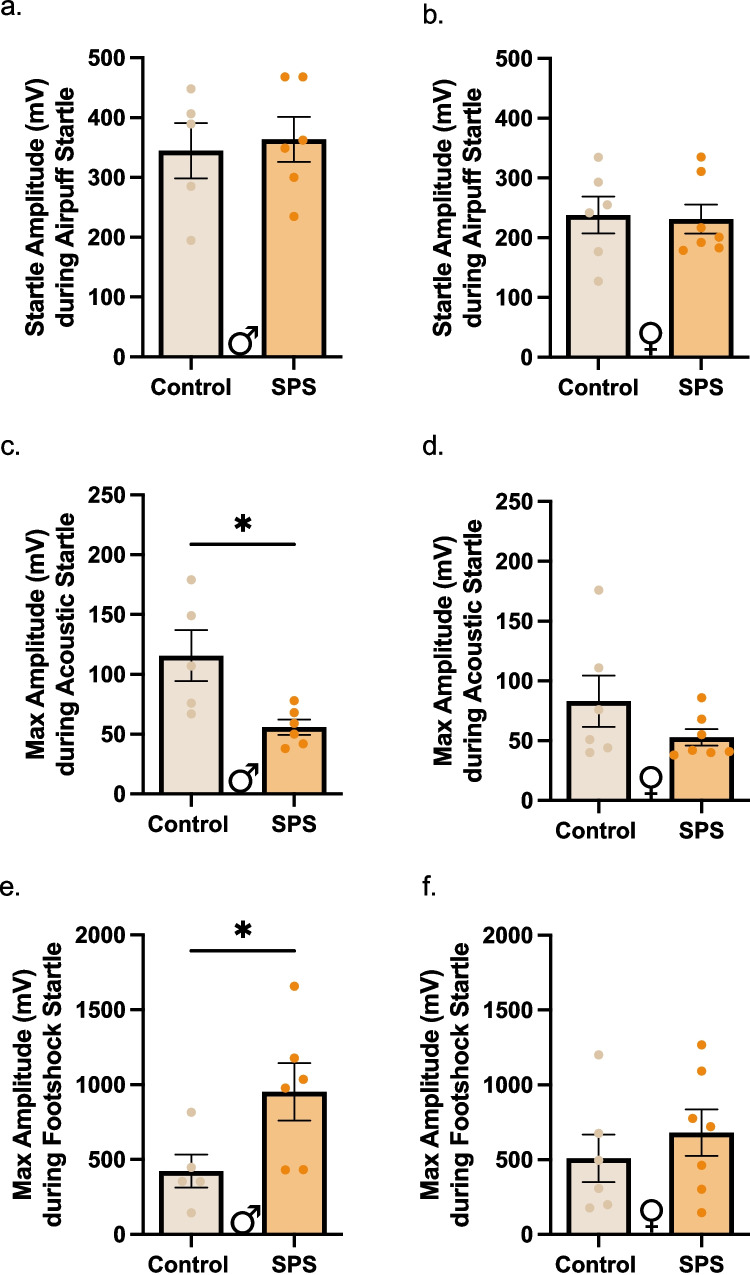
SPS exposure had modality-specific startle effects in male mice. (**a, b**) Average startle response to airpuff presentations was not altered in SPS-exposed male (**a**) and female (**b**) mice. (c, d) There was a decrease in startle response during the first presentation of the acoustic tone (90 dB) in male SPS mice (**c**) but not SPS females (**d**) compared to their control counterparts. (**e**) SPS males exhibited an average increase in startle response elicited during the three presentations of 0.4 mA footshock. (**f**) There were no differences in average startle response in the female SPS mice at 0.4 mA footshock. (* = *p* < 0.05)

Next, we further explored this sex specific NSFT effect by conducting additional tests for negative affect. In addition to anxiety-like phenotypes, traumatic stress increases startle responses to unexpected stimuli in the clinical population (Shalev et al. 2000; Morgan et al. 1995). Thus, to investigate the effect of SPS on startle, male and female mice were exposed to three different sensory stimuli- airpuff, acoustic stimuli, and footshock. In all three tests, we ran pairwise comparisons of treatment groups in male and female mice for the first, novel stimulus presentation, and the average startle response for a given stimulus intensity. The full analysis is depicted in Table 1. Starting with airpuff stimuli, mice received three airpuffs of the same intensity. There were no effects of SPS on first exposure (Table 1) or average startle response in male (Student’s t-test: t_(9)_ = 0.3224, *p* = 0.7545; Fig. 2b) or female mice (Student’s t-test: t_(11)_ = 0.1774, *p* = 0.8625; Fig. 2c). Next, we performed acoustic startle at three different decibels (90 dB, 95 dB, and 105 dB), each presented in three successive trials. During the first exposure (90 dB stimulus), SPS males had lower startle amplitude compared to control males (Student’s t-test: t_(9)_ = 2.913, *p* = 0.0172; Fig. 2e), while no such differences were noted in SPS females (Student’s t-test: t_(11)_ = 1.437, *p* = 0. 1813; Fig. 2f). There was a similar decrease observed in average startle response in male mice at 105 dB stimulus, while no differences in the averages at any decibel in female mice (Table 1) were noted. On footshock startle response, mice were exposed to 3 trials of 6 shock intensities (in this order: 0.2, 0.4, 0.05, 0.6, 0.1, 0.8 mA). There was no effect of SPS on first stimulus presentation (0.2 mA) in male or female mice (Table 1). However, the average startle response of the 0.4 mA shock revealed an increase in SPS males (Student’s t-test: t_(9)_ = 2.265, *p* = 0.0498; Fig. 2h), but not SPS females (Student’s t-test: t_(11)_ = 0.7708, *p* = 0.4570; Fig. 2i). Interestingly, there was a decrease in the average startle response during of SPS females during 0.05 mA shock (Student’s t-test: t_(11)_ = 2.342, *p* = 0.0390). This was not seen in SPS males (Student’s t-test: t_(9)_ = 0.4645, *p* = 0.6534) (Table 1). Overall, these results reinforce the previous observation that males, but not females, exhibit SPS effects on negative affect-like behavior, and these effects are not uniform across tests.

**Table 1:** Statistical comparisons of startle response across stimulus types and intensities in male and female subjects. Max amplitude during the first presentation and average amplitude across intensities were analyzed using independent t-tests. Stimuli included airpuff, acoustic tones (90 dB, 95 dB, 105 dB), and footshock (0.2, 0.4, 0.05, 0.6, 0.1, 0.8 mA). Reported values include t-statistics and p-values for each comparison. Significant results (*p* < 0.05) are bolded

	Males	Females
Max Amplitude during first presentation of each stimuli		
Airpuff	t = 0.2314, p = 0.8222	t = 1.028, p = 0.3262
Acoustic	t = 2.913, **p = 0.0172**	t = 1.427, p = 0.1813
Footshock	t = 0.5270, p = 0.6109	t = 0.3072, p = 0.7650
Average Amplitude during each stimuli intensity		
Airpuff	t = 0.3224, p = 0.7545	t = 0.1774, p = 0.8625
Acoustic		
90 dB	t = 1.498, p = 0.1683	t = 2.084, p = 0.0613
95 dB	t = 0.07092, p = 0.9450	t = 0.4425, p = 0.6667
105 dB	t = 3.053,**p = 0.0137**	t = 0.3302, p = 0.7474
Footshock		
0.2 mA	t = 1.458, p = 0.1790	t = 0.6959, p = 0.5009
0.4 mA	**t = 2.265, p = 0.0498**	t = 0.7708, p = 0.4570
0.05 mA	t = 0.4645, p = 0.6534	t = 2.342, **p = 0.0390**
0.6 mA	t = 0.9588, p = 0.3627	t = 0.3276, p = 0.7494
0.1 mA	t = 1.716, p = 0.1245	t = 0.1421, p = 0.8896
0.8 mA	t = 0.3799, p = 0.7128	t = 0.6807, P = 0.5101

Next, we tested whether individual behavior was consistent across behavioral modalities. To normalize across behaviors, raw behavior data from each test underwent a z-score transformation using the mean of the control group. No consistent individual patterns emerged across behaviors (Supplemental Fig. 1a). From this, we generated a single “emotionality” score for each mouse (Guilloux et al. 2011) taking the average z-score across all behaviors tested. SPS did not alter emotionality scores in either sex compared to their control counterparts (Student’s t-test: males; t_(9)_ = 0.5420, p = 0.6010; females; t_(11)_ = 1.126, *p* = 0.2843; Supplemental Fig. 1b-c).

### Sex-specific analysis reveals no effect of single prolonged stress on ethanol drinking in male or female mice

**Fig. 3:**
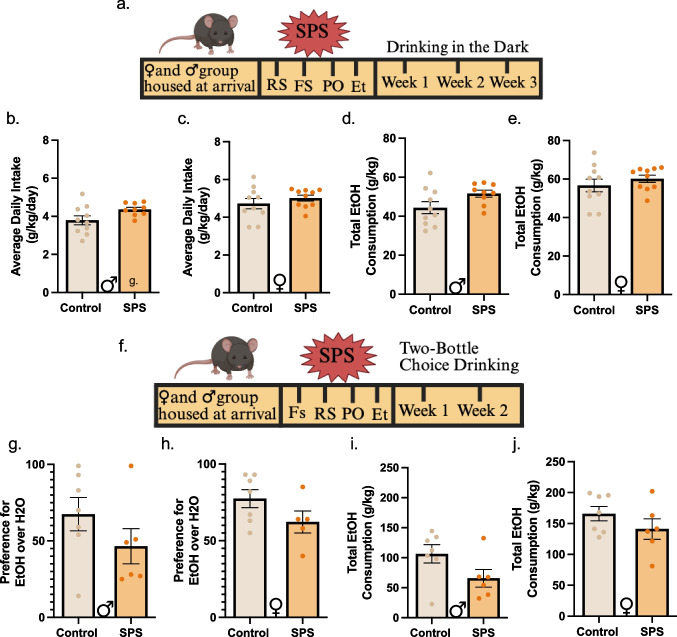
Single Prolonged Stress does not alter ethanol drinking patterns during two volitional ethanol drinking models (a) Experimental Timeline. Mice underwent SPS as described in Figure 1. One week after SPS, mice underwent three cycles of Drinking in the Dark (DID). Each cycle consists of three days of 2h-access to homecage ethanol, one day of 4h access, and three days of no access. (b) (c) Daily Ethanol Intake during DID. SPS did not alter daily average daily ethanol intake in male (b) or females (c). (d) (e) Total Ethanol Consumption during DID. Over the 12 days of DID sessions, comparable amount of ethanol was consumed in both males (d) and females (e). (f). Experimental Timeline. Mice underwent SPS as previously stated. One week after SPS, mice had continuous access to a 10% ethanol bottle and a water bottle for two weeks. (g) (h) Ethanol Preference during 2-BC. Males (g) and females (h) did not alter their preference for ethanol over water after SPS. (i) (j) Total Ethanol Consumption during 2-BC. There were no differences observed in total consumption over the two weeks of access in SPS males (i) or females (j)

Clinically, drinking behavior is impacted by stressful experiences (Magrys and Olmstead 2015; Keyes et al. 2012), yet there is still conflicting preclinical evidence, mainly due to the models, timing of consumption, and sex dimorphisms (Cozzoli et al. 2014; Becker et al. 2011), highlighting the need to refine preclinical models better. To address this gap, we subjected mice to SPS followed by the Drinking in the Dark (DID) procedure, a model for binge-drinking which is a hallmark precursor of AUD (Rhodes et al. 2005). Males and females underwent SPS and three weeks of DID (3 days of 2 h of ethanol access, 1 day of 4 h of ethanol access, and 3 days of no ethanol), during which daily ethanol intake was recorded. SPS did not impact average (Student’s t-test: males; t_(17)_ = 2.096, *p* = 0.513; females; t_(18)_ = 0.9234, *p* = 0.3680; Fig. 3b and c) or total (Student’s t-test: males; t_(17)_ = 1.961, *p* = 0.0665; females; t_(18)_ = 0.9234, *p* = 0.3680; Fig. 3d and e) ethanol consumption throughout the 12 DID sessions.

Next, we evaluated whether the intermittency of ethanol access and binge-like consumption is preventing a SPS phenotype. We tested a cohort of mice in a continuous two-bottle choice (2-BC) paradigm after SPS. Mice received continuous access to ethanol and water for 2 weeks beginning one week after SPS (Fig. 3f). Average preference for ethanol (Student’s t-test: males; t_(11)_ = 1.317, *p* = 0.2146; females; t_(10)_ = 1.646, *p* = 0.1308; Fig. 3g and h) or total consumption of ethanol (Student’s t-test: males; t_(11)_ = 1.901, *p* = 0.0838; females; t_(11)_ = 1.258, *p* = 0.2345; Fig. 3i and j) were similar between groups.

Female mice readily consume more alcohol than males, particularly in volitional drinking models (e.g., Sneddon et al. 2019; Bloch et al. 2020; Rivera-Irizarry et al. 2023). While the primary objective of this study was to examine SPS effects within each sex, we tested whether we could replicate this sex differences in these two drinking models. We performed secondary exploratory analysis to include sex as a covariate. Indeed, ethanol consumption was higher in female in both DID (2-way ANOVA, sex: F _(1, 35)_ = 15.58, *p* = 0.0004; Supplemental Fig. 2a) and 2-BC (2-way ANOVA, sex: F _(1, 22)_ = 21.45, *p* = 0.0001; Supplemental Fig. 2b). An uncorrected Fisher’s LSD post hoc test showed that females increased consumption compared to males and confirmed the sex effect in DID (Control: *p* = 0.0021, SPS: *p* = 0.0293). Interestingly, these analyses revealed DID (2-way ANOVA, treatment: F _(1, 35)_ = 4.082, *p* = 0.0510; Supplemental Fig. 2a) and a significant SPS effect with 2-BC (2-way ANOVA, treatment: F = 5.062, *p* = 0.0348; Supplemental Fig. 2b), although there was no sex x treatment interaction effect in either drinking model (DID F _(1, 35)_ = 0.4773, *p* = 0.4924; 2-BC F _(1, 21)_ = 1.309, *p* = 0.2654). The absence of an interaction between sex and treatment indicates that the treatment effect does not differ significantly between males and females. Thus, post hoc comparisons between sex-treatment combinations are not statistically justified.

### Ethanol consumption is higher during SPS and FC in females with a history of ethanol exposure.

**Fig. 4:**
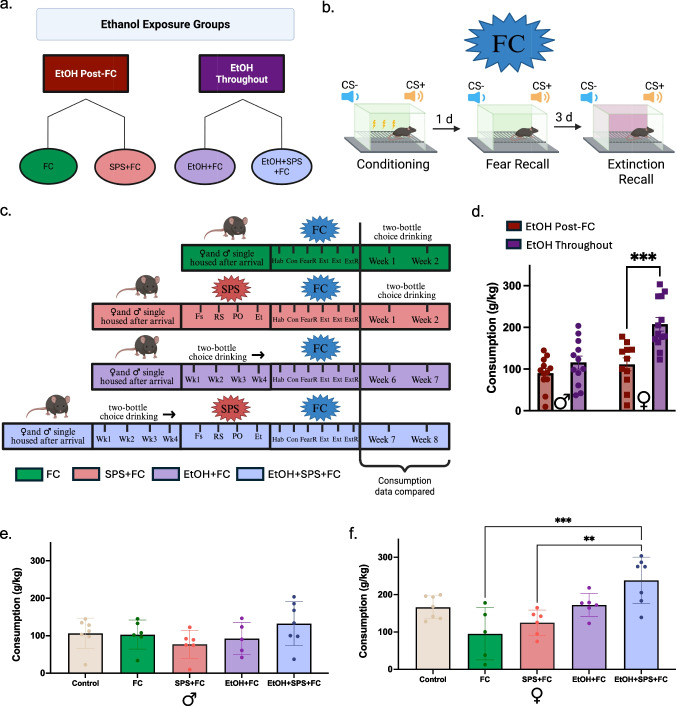
Ethanol consumption patterns are impacted by stress timing, fear learning timing, and a history of ethanol exposure (**a**) Experimental groups categorized by ethanol exposure, EtOH Post-FC (red) and EtOH Throughout (dark purple). Experimental groups within the exposure categories are separated by color: FC (green), SPS + FC (pink), EtOH + FC (purple), and EtOH + SPS + FC (blue). **b** The three main fear conditioning days analyzed from the Fear Conditioning (FC) paradigm. Conditioning is where the CS + tone is paired with a shock (fear cue) while the CS- tone is not (safety cue). Fear Recall is in the same context with the two tones presented, but a shock is not administered. Extinction Recall is in a different context with the two tones presented without a shock after two days of Extinction days. **c** The experimental timeline of each group where consumption data was compared from the last two weeks of 2-BC drinking. **d** Collapsing ethanol consumption by EtOH Throughout and EtOH Post-FC groups for males and females. **e**–**f** Overall ethanol consumption from the last two weeks of 2-BC for males and females. **e** Male ethanol consumption did not change across groups. **f** Female mice with continuous access to ethanol, SPS, and FC significantly increased ethanol consumption compared to those who had ethanol after stressors. Number of mice per group: FC = 6M/5F, SPS + FC = 6M/6F, EtOH + FC = 5M/6F, EtOH + SPS + FC = 7M/7F (* = *p*-value < 0.05, ** = *p*-value < 0.01, *** = *p*-value < 0.001, **** = *p*-value < 0.0001)

Alcohol is used as a coping mechanism to relieve dysregulated stress responses, increasing normal drinking levels (Kube et al. 2023; Herman 1992). One of these dysregulated responses in traumatic-stress patients is inappropriate fear responses that occur in later events, as the number of stress events experienced is associated with greater rates of AUD diagnosis (Keyes et al. 2012). As SPS did not alter subsequent drinking (Fig. 3), we decided to investigate the timing of ethanol exposure and traumatic stress with fear responses and subsequent drinking. To assess the impact of ethanol timing, we implemented two primary conditions: ethanol exposure either before (EtOH Throughout) or after (EtOH Post-FC) behavioral testing (Fig. 4a). In the EtOH Throughout groups, mice had access to ethanol in their home cages before, throughout, and after the behavioral paradigms of fear conditioning (FC) (Fig. 4b) and SPS. Given the consistent effects across DID and 2-BC, we utilized 2-BC as this model is more conducive for minimizing experimenter interventions during the incubation week after SPS.

To examine how volitional drinking changes with the timing of exposure, SPS, and fear responses, four groups were measured for their overall ethanol consumption during the last two weeks of 2-BC (Fig. 4c). The “EtOH Post-FC” groups consisted of the FC group and the SPS + FC group. The “EtOH Throughout” groups consisted of the EtOH + FC group and the EtOH + SPS + FC group (See Fig. 4a-c and Methods for detailed explanation of groups and behavioral procedures). To directly examine exposure time on ethanol consumption, a 2-way ANOVA was conducted on Post-FC and Throughout groups where an interaction effect of exposure time and sex (F_(1, 44)_ = 5.577, *p* = 0.0227) was observed. A post hoc analysis with Šídák's multiple comparisons test showed that females drove this effect (Fig. 4d) (Females EtOH Throughout vs Females EtOH Post-FC, *p* = 0.0003). A 2-way ANOVA comparing group and sex was conducted to assess ethanol consumption. This revealed a main effect of group (F_(3, 40)_ = 8.806, *p* = 0.0001) and sex (F_(1, 40)_ = 15.68, *p* < 0.0001) on ethanol consumption with an interaction effect (F_(3, 40)_ = 2.998, *p* = 0.0419). Post-hoc analysis with Šídák's multiple comparisons test showed that basal sexual-dimorphic drinking behavior is absent when animals are exposed to ethanol after FC and SPS (Supplemental Fig. 2c). It is only when animals are exposed to ethanol before and continuously with SPS or FC that the sex effect remains intact (EtOH + FC, p = 0.0064; EtOH + SPS + FC, *p* < 0.0001) (Supplemental Fig. 2c). Specific sex effects on group and ethanol consumption were examined with a one-way ANOVA for males and females separately (Fig. 4). Males do not show differences in consumption regardless of experimental groups (Fig. 4e). However, there was an effect on consumption in females (F_(4, 26)_ = 7.939, *p* = 0.0003). A post-hoc analysis with Tukey’s multiple comparisons test showed that the EtOH + FC + SPS group has increased consumption compared to the EtOH Post-FC groups (FC vs EtOH + SPS + FC, *p* = 0.0002; SPS + FC vs EtOH + SPS + FC, *p* = 0.0019, Fig. 4f). Overall, these findings suggest that innate patterns of ethanol consumption are shaped by prior stress and fear responses, with increased drinking dependent on the timing of ethanol exposure, affecting females particularly.

### SPS and ethanol exposure disrupt the differentiation between safety and fear cues.

**Fig. 5:**
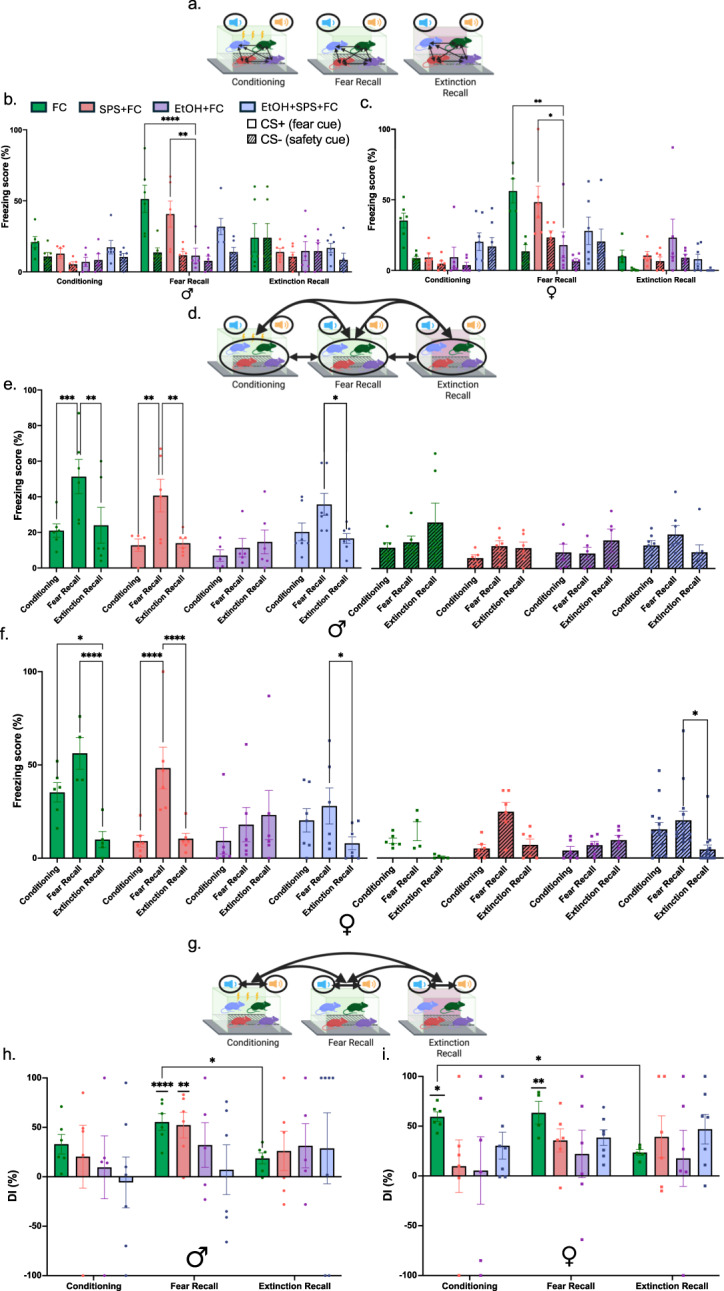
Prior traumatic stress and ethanol exposure disrupts fear differentiation between fear and safety cues (**a**) Graphic showing comparisons in panels b-c which looks at normalized freezing scores towards the safety cue (CS-, blue) and fear cue (CS + , yellow) between groups, described in Fig. 4, on each fear conditioning day. (b-c) Male (**b**) and female (**c**) freezing scores towards the CS + and CS- between the groups on each fear conditioning day. The EtOH + FC group in both males and females froze less towards the fear cue on Fear Recall day compared to the control (FC) and SPS + FC groups. **d** Graphic showing comparisons in panels e–f which compares freezing scores towards cues across days for each group (not comparing other groups to each other). (e–f) Male (**e**) and female (**f**) freezing towards cues on each day. In males and females, both the control and SPS + FC groups showed significant differences in their responses only towards fear cues across days. The EtOH + FC group did not alter freezing towards both cues across the days. The ethanol-stressed group (EtOH + SPS + FC) showed decreased freezing towards both cues from Fear Recall to Extinction Recall day. Females (**f**) also show decreased freezing towards the safety cue from Fear Recall to Extinction Recall day. **g** Graphic showing comparisons in panels h-i which compares freezing between cues on and across days with normalized freezing scores showing significance on the day and Discrimination Index (DI) scores showing significance across days. **h** Male freezing between cues on and across days. The control and SPS + FC groups showed increased freezing towards fear cues on Fear Recall day. DI scores decreased in the control group from Fear Recall to Extinction Day. **i** Female freezing between cues on and across days. The control group had increased freezing towards fear cues on Conditioning and Fear Recall day. DI scores decreased in the control group from Conditioning Day to Extinction Day for females. Neither ethanol-exposed groups in both males and females showed a difference in freezing between the cues across all days. All groups besides the controls did not change their DI scores across the days. Number of mice per group: FC = 6M/5F, SPS + FC = 6M/6F, EtOH + FC = 5M/6F, EtOH + SPS + FC = 7M/7F (* = *p*-value < 0.05, ** = *p*-value < 0.01, *** =* p*-value < 0.001, **** = *p*-value < 0.0001)

Traumatic stress disrupts fear memory and fear responses (Schein et al. 2021), which can be explored preclinically by utilizing SPS and FC in mice (Yamamoto et al. 2008). Moreover, alcohol is used to cope with these disrupted responses and necessitates further exploration in preclinical models. We next examined the effect of prior stress and ethanol exposure on fear responses (i.e., freezing) from the groups described above (Fig. 4a-c): FC (control), SPS + FC, EtOH + FC, and EtOH + SPS + FC. Freezing scores towards fear cues and safety cues are normalized to each animal’s habituation day to ensure freezing is due to conditioning and not because of tone sensitivity. A two-way ANOVA comparing group and fear conditioning day was conducted for each sex separately to assess specific sex effects on freezing across the three main FC days: Conditioning, Fear Recall, and Extinction Recall (Fig. 5). There were main effects of group (Males, F_(2, 113)_ = 8.033, *p* = 0.0005; Females, F_(2, 118)_ = 17.74, *p* < 0.0001) and fear conditioning day (Males, F_(7, 113)_ = 6.473, *p* < 0.0001; Females, F_(7, 118)_ = 5.681, *p* < 0.0001), and an interaction effect of group x day (Males, F_(14, 133)_ = 2.085, *p* = 0.0176; Females, F_(144, 118)_ = 2.675, *p* = 0.0020). Post-hoc analysis with Tukey's multiple comparisons test allowed for further comparison of differences in freezing scores towards the cues between groups on each day (Fig. 5a), across days for each group (Fig. 5d), and between cues on each day (Fig. 5g).

Comparing freezing towards cues between groups on each day (Fig. 5c-b) showed that both sexes displayed similar responding towards safety cues between groups on each day. Additionally, for freezing towards fear cues on Fear Recall day the EtOH + FC group had decreased freezing compared to non-ethanol exposed groups for both sexes (Males, FC CS + vs EtOH + FC CS + , *p* < 0.0001; Females FC CS + vs EtOH + FC CS + , *p* = 0.0031; Males, SPS + FC CS + vs EtOH + FC CS + , *p* = 0.0095; Females, SPS + FC CS + vs EtOH + FC CS + , *p* = 0.0139). The group effects on each day (Fig. 5c-b) suggest that continuous ethanol exposure significantly reduces fear responses towards fear cues on Fear Recall day in both sexes.

Freezing scores were also compared across the days for each group with the same post-hoc analysis (Fig. 5e-f). Controls, the FC group, both males (Fig. 5e) and females (Fig. 5f), showed decreased freezing towards the fear cue on Extinction Recall compared to Fear Recall (Males, FC Fear Recall CS + vs FC Extinction Recall CS + , *p* = 0.0018; Females, FC Fear Recall CS + vs FC Extinction Recall CS + , *p* < 0.0001). Overall, this suggests that the paradigm induces fear learning in our control animals. The addition of SPS (SPS + FC group) also induced sufficient fear learning in both males and females (Males, SPS + FC Conditioning CS + vs SPS + FC Fear Recall CS + , *p* = 0.024; Female, SPS + FC Fear Recall CS + vs SPS + FC Extinction Recall CS + , *p* < 0.0001). The EtOH + FC group in both males and females did not change fear responding towards both cues across the days (*p* > 0.05), confirming Fig. 5c-b results that ethanol successfully dampens fear responding over the three main phases of fear learning. The EtOH + SPS + FC group also showed successful fear learning with decreased freezing from Fear Recall to Extinction Recall days toward fear cues in both sexes (Males, EtOH + SPS + FC Fear Recall CS + vs EtOH + SPS + FC Extinction Recall CS + , *p* = 0.0241; Females, EtOH + SPS + FC Fear Recall CS + vs EtOH + SPS + EtOH Extinction Recall CS + , *p* = 0.0351) and safety cues in females (Females, EtOH + SPS + FC Fear Recall CS- vs Extinction Recall CS-, *p* = 0.0239). This suggests that prior SPS paired with continuous access to ethanol enhances fear extinction by reducing fear responses toward fear cues, and both cues for females, in non-fearful environments.

To quantify how accurately animals differentiated between fear and safety cues, a Discrimination Index (DI) was calculated with the normalized freezing scores (described in the Methods) to measure the performance of each animal across the three fear conditioning days (Fig. 5h-i). Positive values indicate more freezing towards CS + (fear cue), while negative values indicate more freezing towards CS- (safety cue), and values near zero mean insufficient discrimination. The significance of freezing scores between cues for each group was determined with the same Tukey’s multiple comparisons test on normalized freezing scores and are shown over the appropriate group discrimination index (Fig. 5h-i). For males, the FC and SPS + FC groups showed increased freezing towards the fear cue compared to the safety cue on Fear Recall day (Males, FC Fear Recall CS + vs FC Fear Recall CS-, *p* < 0.0001; SPS + FC Fear Recall CS + vs SPS + FC Fear Recall CS-, *p* = 0.0058; Fig. 5h). For females, the FC group showed increased freezing towards the fear cue on both Conditioning and Fear Recall days (Females, FC Conditioning CS + vs FC Conditioning CS-, *p* = 0.0492; FC Fear Recall CS + vs FC Fear Recall CS-, *p* = 0.0023; Fig. 5i). To compare DI scores on group and fear conditioning days, a mixed-effects model (REML) was used, and no significant fixed effects emerged. However, when examining each group effect, only the control group showed a different level of discrimination between cues across the days for both sexes. In males, there was decreased discrimination between the Fear Recall and Extinction Recall days (FC Fear Recall vs FC Extinction Recall, *p* = 0.0207). In females, there was decreased discrimination between the Conditioning and Extinction Recall days (FC Conditioning vs FC Extinction Recall, *p* = 0.0157). This suggests that both males and female controls were successfully conditioned to the correct stimuli (CS +) and showed signs of extinction with decreased discrimination on the last day. However, males and females that were exposed to prior stress, ethanol access, or both did not show this difference in their DI scores. This indicates that prior stress and ethanol access disrupted the adjustment of cue discrimination across the different conditioning days.

## Discussion

This study examined the validity and effectiveness of using SPS in mice to study traumatic stress effects on negative affect, fear memory, and ethanol consumption. The primary goal was to expand the utility of SPS broadly, while considering sex specific effects, allowing further investigations to explore the neurobiology of traumatic stress and its interactions with PTSD-like phenotypes and alcohol-related behaviors. Historically, much of the SPS literature has focused on the effects it has on male rats. We address this gap by including both male and female mice. Our work demonstrates that SPS induces negative affect-like phenotypes in male mice during specific behavioral modalities, whereas female mice were unaffected by SPS. Female mice were more susceptible to the timing of ethanol access with SPS, exhibiting increased consumption compared to their counterparts that had ethanol exposure afterwards. The sex specific differences exposed by SPS-induced negative affect and temporal ethanol consumption reinforce the importance of investigating sexual dimorphisms in preclinical research. Furthermore, SPS disrupted the flexibility in fear and safety cue discrimination across different environments in both males and females, mirroring the dysregulated fear responses seen in PTSD clinical populations. Overall, the findings in this paper highlight the use of SPS in mice to explore sex-dependent PTSD symptomology and subsequent alcohol-related behaviors.

### Single prolonged stress selectively induces negative affect and produces behavior-specific effects in male mice

While many report increases in anxiety-like phenotypes after SPS using EPM in rats (Fang et al. 2018; Serova et al. 2019) and a few in mice (Lin et al. 2020; Kurilova et al. 2023; Zhao et al. 2025), we did not observe SPS effects in either sex during EPM in our mice (Fig. 1d-e). Continuous re-exposure to at least one component of SPS (Kurilova et al. 2023) or an additional stressor, like footshock (Zhao et al. 2025), induces EPM effects in mice, suggesting that a more potent experience of stress may be needed for mice to display changes in negative affect-like phenotype on EPM. Interestingly, in NSFT, SPS males showed an increased latency to eat, whereas females did not (Fig. 1f-g), which is in agreement with recent reports in rats (Pitcairn et al. 2024). This is particularly intriguing as NSFT probes for an anxiety-like phenotype by leveraging hyponeophagia (Blasco-Serra et al. 2017), which males may be more sensitive to. It is important to note that, on average, control females took 2 × more time to approach and eat the food pellet compared to the control males. It is plausible that the high latency in control females created a ceiling effect in females.

Given the well-characterized link between traumatic stress and social deficits (Charuvastra 2008), we incorporated this test into our study. Although less commonly used in SPS, Ben-Azu et al. (2024) noted a decrease in social preference during social interaction (SI) in Swiss mice. SPS did not change social behavior in C57BL/6 J mice, regardless of sex (Fig. 1b-c). While EPM, NSFT, and SI are traditional approach-avoidance tests associated with negative affect-like phenotypes, startle response testing assesses non-locomotor-based phenotypes of negative affect.

Increased startle is widely implicated in stress (Kozarić-Kovačić et al. 2011; Herten et al. 2016; Ray et al. 2009; Stout et al. 2021), encouraging further investigation in preclinical models. Here, SPS did not alter average startle response to airpuff stimuli (Fig. 2a-b). There was an increase in average startle response in SPS males during 0.4 mA footshock, but not females (Fig. 2e-f). We postulate that an optimal footshock level is necessary to induce startle, reflecting an inverted-U curve relationship where levels too low and levels too high were insufficient to produce a startle response. We speculate that the diversion in the direction of effects noted in SPS males during acoustic and footshock is due to the modality and saliency of each stimulus. While footshock induces a sensory pain response that alters and strengthens its saliency (Bonnet and Peterson 1975; Legrain et al. 2011), acoustic stimulation utilizes auditory sensory input to induce the startle response. Though stress can increase pain sensitivity (Crettaz et al. 2013; Vachon-Presseau et al. 2013; Han 2022; Abouhaar and Serrano 2024), this has not been directly tested with SPS. Follow-up studies can investigate SPS’s effects on perception and sensitivity to pain using assays like the Von Frey Test. Furthermore, footshock can serve as a stressor in and of itself (Wu et al. 2020); thus, its ability to induce an SPS effect is consistent with the theory that additional stressors, in conjunction with SPS, are necessary to produce negative affect-like phenotypes in mice.

While emotionality scores were comparable to controls for both SPS males and females, it is worth considering that SPS is an acute stressor that relies on a multimodal approach encompassing physical, predatory, and psychosocial stress. Results indicate that female mice were not affected by the combination of multiple modalities of stress in an acute manner, similar to the resiliency female rats display after SPS (Keller et al. 2015). The modality of the behavior must also be considered as males showed robust SPS effects in a hyponeophagia based assay (NSFT) whilst the SPS females were unaffected. This pattern is further evidenced by SPS males exhibiting a heightened average startle response to 0.4 mA footshock, while females demonstrated responses comparable to control females across all footshock intensities, apart from a reduced average startle response at the lowest intensity. While both could be attributed to estrogen levels (Wei et al. 2014), future studies will further explore how these stressors individually alter behavior in males and females and whether repeated exposure to SPS, combined with the inclusion of an additional stressor, induces negative affect-like phenotypes in both sexes and give rise to resilient and susceptible subpopulations.

### Sex-specific analysis reveals no effect of single prolonged stress on ethanol drinking in male or female mice

Given the lack of consensus on the relationship between stress exposure and alcohol consumption (Becker et al. 2011; Delis et al. 2015; Piggot et al. 2020), we investigated the impact of SPS on volitional drinking behavior in C57BL/6 J mice, a relationship that has not been thoroughly examined. SPS did not alter ethanol consumption in either the binge model or the continuous access model (Fig. 3b-j). While our primary analysis aimed to uncover the effect of SPS on ethanol consumption in males and females, secondary analysis allowed for sex differences to be investigated. We replicated the well-documented higher ethanol consumption patterns in female mice (Sneddon et al. 2019; Bloch et al. 2020; Rivera-Irizarry et al. 2023) in both DID and 2-BC, regardless of stress (Supplemental Fig. 2a-b). Interestingly, while SPS did not change basal sex differences in drinking patterns, considering sex as a covariate led to an overall decrease in ethanol consumption during 2-BC and a trending increase during DID. This hints at a potentially interesting interaction between stress and ethanol. Further studies should investigate drinking architecture to identify any underlying individual differences, such as number of licks, lick duration, bout of licks, in males and females following SPS.

### The timing of ethanol exposure is necessary for stress-induced drinking changes

PTSD patients have challenges in extinguishing fear memories, exacerbating stress responses in everyday life (Schein et al. 2021) that could lead towards ‘self-medicating’ (Khantzian 1997) with substances such as alcohol, increasing the risk of AUD. Given this relationship, we examined the timing of ethanol exposure relative to stress, with SPS, and fear learning, with FC, on ethanol consumption with four groups categorized as “EtOH Post-FC” and “EtOH Throughout” (Fig. 4a-c). Since SPS requires an undisturbed week for stress consolidation (Fang et al. 2018), two-bottle choice was used as the drinking model to minimize experimenter intervention. The EtOH Post-FC category encompassed groups who received ethanol after behavioral procedures: FC and SPS + FC groups. The EtOH Throughout category had groups who received ethanol prior and continuously throughout behavioral procedures: EtOH + FC and EtOH + SPS + FC groups (See Fig. 4a-c and Methods). EtOH Post-FC and EtOH Throughout groups showed that females increased consumption with prior and continuous access to ethanol (Fig. 4d). This sex effect is further displayed by the EtOH + FC and EtOH + SPS + FC groups restoring the sex effect that disappears in EtOH Post-FC groups (Supplemental Fig. 2c). The EtOH + SPS + FC group only in females increased overall ethanol consumption compared to groups exposed to ethanol afterwards (Fig. 4f). This suggests that in females, the combination of SPS and FC is sensitive to the timing of ethanol exposure, driving increased ethanol consumption. Overall, females are more susceptible to the timing of ethanol exposure and access, especially when paired with both SPS and FC. Interestingly, FC, SPS, and timing of exposure paired with SPS and/or FC did not change consumption when compared to our controls in both males and females (Fig. 4e-f). This finding aligns with previous literature, which suggests that animals undergoing stress prior to ethanol exposure do not exhibit differences in volitional ethanol consumption compared to controls (Piggot et al. 2020; Hackleman et al. 2023; Pitcairn et al. 2024; Mayberry et al. 2025). This suggests that the effects of stress on ethanol consumption may be more insightful by exploring the timing of ethanol exposure and comparing stressed groups. This approach in preclinical models is more translational to the human population since about 79% of Americans reported alcohol use in their lifetime (SAMHSA 2023) and 70% of Americans reported stress symptoms in the past month (APA 2023), indicating that alcohol history prior to stress events is most likely. Moreover, an alternative hypothesis in explaining AUD and traumatic stress comorbidity is that individuals with substance misuse are vulnerable to the effects of traumatic events, further perpetuating the addiction cycle (Cottler et al. 1992). Incorporating prior and continuous ethanol access with stressors enables exploration of these clinically relevant circumstances. Overall, we demonstrate that ethanol consumption increases in response to the combination of stress, fear learning, and ethanol exposure. This suggests that having prior and continuous access to ethanol with stress increases ethanol volitional drinking, especially in females, compared to exposure afterwards. These findings are consistent with studies that explore how earlier exposures to ethanol lead to susceptibility in developing AUD (Shnitko et al. 2016; Fernandez et al. 2016). Future studies can determine if the amount and type of drinking (e.g., binge-like) before and during a traumatic stressor impacts fear learning, affective behavior, and subsequent drinking.

### SPS and ethanol exposure disrupt the differentiation between safety and fear cues

To explore the relationship between traumatic stress and ethanol exposure on fear responses, freezing behavior was analyzed during FC with two auditory tones, a fear cue (CS +) that is paired with a shock, and a safety cue (CS-) which is not paired with a shock, on three distinct days: Conditioning, Fear Recall, and Extinction Recall. The groups as explained above (Fig. 4a-c and Methods) that underwent fear conditioning (FC, SPS + FC, EtOH + FC, and EtOH + SPS + FC) were used. In both males and females, the FC and SPS + FC groups had successful fear learning when comparing cues on each day (Fig. 5d-f). Despite their success, SPS + FC male and female mice failed to adjust their Discrimination Index (DI) across the days, suggesting that with SPS, they similarly discriminate between the fear cue and the safety cue even on the Extinction Recall day (Fig. 5h-i). Overall, SPS heightens discrimination even after extinction periods, disrupting the ability to adapt fear discrimination in different contextual environments and with repeated extinction sessions. These results align with previous studies, which have concluded that when rats are exposed to stress before a reward-fear-safety cue discrimination task, they exhibit increased freezing towards reward and safety cues compared to non-stressed animals (Woon et al. 2020). This further supports the notion that prior stress modulates the misinterpretation of what is perceived as fearful, safe, and even rewarding. In our paradigm, SPS affected the adjustment of fear cue discrimination on Extinction day, misinterpreting the extinction of fear cues commonly seen in PTSD patients.

Additionally, ethanol disrupts fear conditioning in a dose-dependent manner for both mice and rats when injected with ethanol 15 min before Conditioning and Fear Recall days (Gould 2003; Melia et al. 1996). In the present study, the EtOH + FC group in both males and females exhibited lower freezing towards fear cues on the Fear Recall day compared to controls (FC group) and SPS (SPS + FC group) groups (Fig. 5b-c). Consequently, this overall decrease in freezing altered their DI scores so that ethanol-exposed mice failed to distinctly discriminate between fear and safety cues (Fig. 5h-i). This suggests that FC with continuous access to ethanol drives mice to respond towards fear cues similarly to how they would towards safety cues. Interestingly, the EtOH + SPS + FC group in both males and females showed decreased freezing on Extinction Recall compared to Fear Recall with females even lowering fear responses towards safety cues (Fig. 5e-f). The EtOH + SPS + FC group’s decreased freezing emphasizes the relieving properties of ethanol on SPS, making ethanol highly effective in responding to cues in safe-associated environments for groups exposed to SPS. However, the disrupted learning could also be considered maladaptive, potentially leading to increased ethanol use that can be harmful, perpetuating the cycle of AUD. Overall, prior stress and ethanol exposure disrupt the differentiation between fear and safety cues, where SPS increases responses towards safety cues on Fear Recall day. In contrast, ethanol minimizes overall fear responses towards both cues. This behavior translates well to the self-medicating hypothesis (Khantzian 1997), where animals exposed to SPS express inabilities to adjust fear discrimination, while animals with SPS and access to ethanol have reduced fear responses towards both cues in safe environments.

## Conclusion

The present study highlights the importance of leveraging a multimodal preclinical model of traumatic stress, SPS, for mice. It emphasizes the value of investigating distinct temporal windows of ethanol exposure to further understand stress-induced ethanol consumption and symptomology. Overall, the usage of SPS as a preclinical model of traumatic stress allowed for intricate sex specific effects to emerge and for the examination of complex behavioral responses, such as fear discrimination, revealing a valuable modality for studying PTSD-like phenotypes in mouse models and additionally, exploring different time windows of ethanol exposure allowed for differences in consumption to emerge due to traumatic stress. This approach translationally incorporates prior alcohol exposure and access commonly seen in clinical populations and may offer optimal conditions to study stress-induced drinking in preclinical models. Future studies will include SPS to investigate the neural circuitry driving traumatic stress-induced responses and ethanol consumption, leading towards the development of individualized therapeutics for stress disorders and AUD.

## Supplementary Information

Below is the link to the electronic supplementary material.

Supplementary file1 (DOCX 326 KB)

## Data Availability

Upon publication, all data will be deposited following the NIH Data Management and Sharing Policy. All raw data from this study will be made available upon written request to the corresponding author.
