# Supplementary material for: Single prolonged stress in mice: interactions between alcohol drinking, negative affect, and fear learning
Source: Psychopharmacology (Berl). 2025 Nov 24;243(5):1029–48. doi: 10.1007/s00213-025-06968-8 (PMC13242430; doi:10.1007/s00213-025-06968-8)
Supplement: Supplementary file 1 — Supplementary file1 (DOCX 326 KB) [file 213_2025_6968_MOESM1_ESM.docx]

**Single Prolonged Stress in Mice: Interactions Between Alcohol Drinking, Negative Affect, and Fear Learning**

Aditi B. Buch^1*^, Ava L. Shipman^1*^, Nicolas J. Azzarello^1^, Annie W. Zhou^1^, Samuel W. Centanni^1#^

*These authors contributed equally

*Psychopharmacology*

^#^Corresponding author

Samuel W Centanni, PhD

Piedmont Triad Community Research Center

115 S Chestnut Street

Winston-Salem, NC 27012

Phone: (336) 716-8567

Email: [Samuel.Centanni@advocatehealth.org](mailto:Samuel.Centanni@advocatehealth.org)

**Supplemental Figure 1 Quantification of Emotionality Scores reveals no effect of SPS exposure in males or females** (a) Representative heatmap displaying z-scores (z>0 or z<0) normalized to the mean of the control group for each animal during each behavior. (b) (c) Emotionality Score. Averaged z-scores across all behavioral parameters did not reveal any significant changes in emotionality in SPS males (a) or females (b).

**Supplemental Figure 2 Sex comparison of total ethanol consumption across two volitional ethanol drinking models, stress, and fear** (a) Females in both control and SPS groups consumed more ethanol than their male counterparts during the 12 sessions of DID. (b) Similarly, in a continuous 2-BC paradigm, total ethanol consumption was altered as a result of stress and sex. (c) 2-BC ethanol consumption with different exposure times, stress, and fear experiences. There is a lack of sex difference when mice are exposed to ethanol after fear and stress experience. The sex difference in consumption is restored with ethanol exposure prior to fear and stress experiences. (* = p-value < 0.05, ** = p-value < 0.01, *** = p-value < 0.001, **** = p-value < 0.0001).
